# Supplementary material for: Genetic Diversity and Possible Origins of the Hepatitis B Virus in Siberian Natives
Source: Viruses. 2022 Nov 7;14(11):2465. doi: 10.3390/v14112465 (PMC9693834; doi:10.3390/v14112465)
Supplement: Supplementary file 1 [file viruses-14-02465-s001.zip › Supplementary Figure S1. ML-tree of 1053 studied HBV strains.pdf]

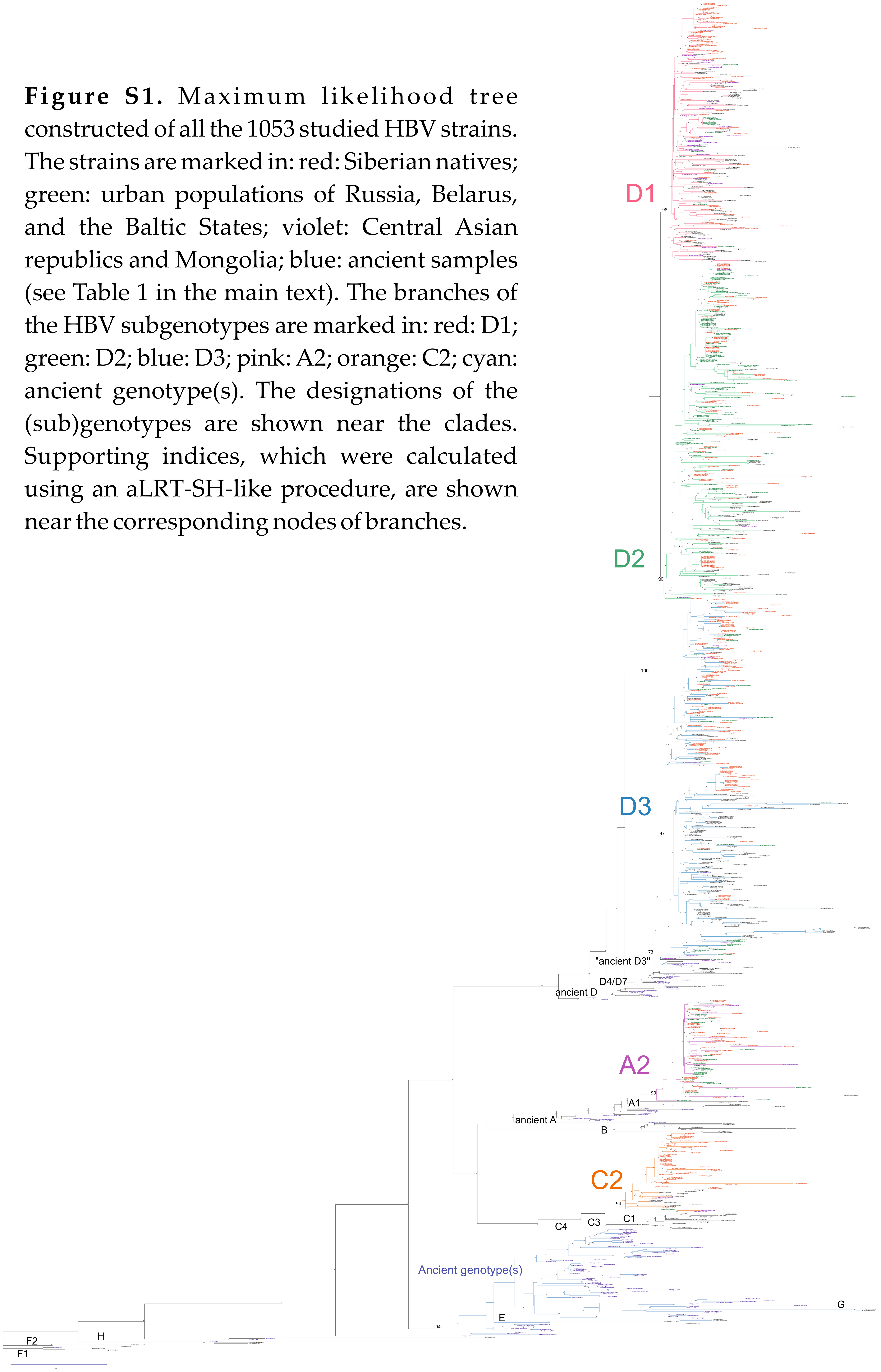

Figure S1. Maximum likelihood tree constructed of all the 1053 studied HBV strains. The strains are marked in: red: Siberian natives; green: urban populations of Russia, Belarus, and the Baltic States; violet: Central Asian republics and Mongolia; blue: ancient samples (see Table 1 in the main text). The branches of the HBV subgenotypes are marked in: red: D1; green: D2; blue: D3; pink: A2; orange: C2; cyan: ancient genotype(s). The designations of the (sub)genotypes are shown near the clades. Supporting indices, which were calculated using an aLRT-SH-like procedure, are shown near the corresponding nodes of branches.
