# Supplementary material for: Genetic Diversity and Possible Origins of the Hepatitis B Virus in Siberian Natives
Source: Viruses. 2022 Nov 7;14(11):2465. doi: 10.3390/v14112465 (PMC9693834; doi:10.3390/v14112465)
Supplement: Supplementary file 1 [file viruses-14-02465-s001.zip › Supplementary Table S1. Anthropological, genetic and linguistic gropus in Siberia.pdf]

Table S1. Anthropological, genetic and linguistic summary for the studied Siberian native groups.

Group	Conventional anthropological genesis [64]	Dominant haplogroups [77-81]	Languages (from family to local dialect) [82]
**Altaians** (Southern)	Turks and Siberian Mongols; ~ 1st millennium BCE	**Y-chromosome:** R1a1 (60%), N (10%), O (6%), D (5%), Q (4%), C3 (2%)<br>**Mt DNA:** C, U, D	Turkic → Common Turkic → Kipchak → East Kipchak → Southern Altai
**Teleuts**	Northern sub-ethnos of Altaians; ~ 4th century CE	**Y-chromosome:** R1a1 (34%), R1b (31%), N1c1 (26%), C3d (5%)	Turkic → Common Turkic → Kipchak → East Kipchak → Southern Altai
**Kazakhs** (Altaian)	Turks, South-Siberian Mongoloid tribes, Middle Eastern Arabic; ~13–15 centuries CE	**Y-chromosome:** C2 (43%), G1 (12%), R1a (11%), R1b (6%), J2 (6%), O (5%), N1 (5%)	Turkic → Common Turkic → Kipchak → South Kipchak → Kazakh
**Tuvans**	Middle Eastern Turk tribes, Xiongnu (or Hunnic, ancient Chinese Mongoloid tribes); ~ 1st millennium BCE	**Y-chromosome:** N1b (25%), N1c1 (19%), C3 (16%), Q (14%), R1a1 (12%), R1b (3%)	Turkic → Common Turkic → South Siberian Turkic → Sayan → Tuvinian
**Khants**	Ugric, circumpolar paleo-Mongoloids (ancient aborigines), ~ 4th century BCE – 2nd century CE	**Y-chromosome:** N1b (57%), Q1a3 (21%), R1a1 (14%), N1c1 (7%)	Uralic → Khantyic → West Khanty → Northern Khanty → Suryskarer Khanty
**Komi**	Finno-Ugric; ~ 1st millennium BCE	**Y-chromosome:** R1a1 (60%), N1 (33%), R1b1 (5%), J2 (2%)<br>**Mt DNA:** H, U, T	Uralic → Permian → Komi → Komi-Zyrian
**Nenets**	Samoyedic (Uralic Ugric), circumpolar paleo-Mongoloids (ancient aborigines), ~ 1st–2nd millennia CE (?)	**Y-chromosome:** N1b (56,8%), N1c (40,5%), R1a1 (5%), Q (1,4%)	Uralic → Samoyedic → Enets-Nenets → Nenets → Tundra Nenets
**Selkups**	Eastern Samoyedic (Ugric), ~ 6th century CE	**Y-chromosome:** Q (66,4%), R1a1 (19%), N1b (6,9%), R1b (6,1%)	Uralic → Samoyedic → Kams-Selkup → Selkup
**Kets**	Yenisean (from Yenisei River) paleo-Mongoloids, Samoyedic (Ugric); ~ 1st–2nd millennia CE	**Y-chromosome:** Q1a3 (84%), N1c1 (8%), N1b (4%), R1a1 (4%)<br>**Mt DNA:** U, M, C, H, A	Yenisean → Northern Yeniseian
**Nganasans**	Polar Samoyedic; ~ 18th century CE	**Y-chromosome:** N1b (92%), C (5%), O (3%)	Uralic → Samoyedic → Nganasan
**Dolgans**	Northwestern sub-ethnos of Yakuts (see below); ~ 19th century CE	**Y-chromosome:** C3c (35%), N3 (25%), E (5%)<br>**Mt DNA:** C (55%), D (18%), F (5%)	Turkic → Common Turkic → North Siberian Turkic → Dolgan
**Yakuts**	Evenks (ancient Tungus and paleo-Mongoloid derived tribes), Turks; ~10th –16th centuries CE	**Y-chromosome:** N3a (89%), R1a1 (3,7%), N2 (2,75%), C3xV77 (1,8%), C3c (1,8%)<br>**Mt DNA:** C, D	Turkic → Common Turkic → North Siberian Turkic → Sakha
**Buryats**	Northern Mongols (Xiongnu-derived peoples after later Uighur [Eastern Turkic] influence); ~6th –12th centuries CE	**Y-chromosome:** N1 (48%), C3 (40%), R1a1 (4%)<br>**Mt DNA:** D, C	Mongolic → Eastern Mongolic → Oirat-Khalkha → Khalkha-Buriat → Buriat → Russia Buriat
**Chukchi**	Northeastern paleo-Mongoloids (likely share common ancestors with Native Americans); ~4th –3rd millennia BC	**Y-chromosome:** N1c1 (61%), Q (24%), C3 (15%)<br>**Mt DNA:** A, C, D	Chukotko-Kamchatkan → Chukotian → Chukchi
